# Editorial: Waist-to-height ratio is a simple tool for assessing central obesity and consequent health risk

**DOI:** 10.3389/fnut.2023.1277610

**Published:** 2023-09-26

**Authors:** Jimmy Chun Yu Louie, Abraham Wall-Medrano

**Affiliations:** ^1^Department of Nursing and Allied Health, School of Health Sciences, Swinburne University of Technology, Melbourne, VIC, Australia; ^2^Instituto de Ciencias Biomédicas, Universidad Autónoma de Ciudad Juárez, Ciudad Juárez, Chihuahua, Mexico

**Keywords:** anthropometry, central obesity, adiposity, waist-to-height ratio, preventive health

Since the dawn of the study of the human figure (shape and dimensions), science has consistently made progress in establishing its connection to health and physiologic homeostasis. In Vitruvius' *Ten Books of Architecture* (30–20 BC), the perfect anthropometric harmony that keeps each body part with each other and the height of a person was recognized as the *Divine Golden Section* [Phi (φ) constant = 1.618], a fact perfectly illustrated in Leonardo da Vinci's drawing *e proporzioni del corpo umano secondo Vitruvio*, or *The Vitruvian Man* (1), a nude man facing forward and surrounded by a square and a superimposed circle, proposing for the first time a connection between ideal human body proportions and overall health (2). The Quetelet's index [body mass index (BMI; kg.m^2^) = weight (horizontal axis). height^2^ (vertical axis)^−1^] dates to the nineteenth century and gained popularity as a measure of body fatness between 1970 and 1980 (3); interestingly, many reports state that the φ constant fits almost perfectly with non-overweighted (BMI < 25 kg.m^2^) human bodies (2). However, the importance of body fat compartmentalization instead of overall obesity (BMI) for assessing cardiometabolic health risks began to be recognized 50 years ago, and several anthropometric indicators of central adiposity emerged in the early 80s as better predictors of cardiometabolic risk (4).

Globesity has become the most important public health burden. According to the *World Obesity Federation*, one out of five (women) or seven (men) people will be living with obesity by 2030, a trend that failed to achieve the 2025 WHO target to halt the rise in obesity at the 2010 level (5). Such an unstoppable trend is accompanied by an exponential increase in the global prevalence of cardiometabolic diseases and the increment in disability-adjusted life-years (DALYs), especially in low-to-middle sociodemographic index regions (6). This epidemiological scenario supports the urgent need for simple and reliable but mostly sensitive and validated anthropometric indices for screening body fat-related morbidity/mortality. As stated before, while BMI has been extensively used to estimate whole-body fatness, it does not discriminate on the location of fatty depots, a fact particularly important when studying disease phenotypes related to regional–temporal adiposity (7). Waist circumference (WC) and the waist-to-hip ratio (WHR) are commonly used to evaluate abdominal obesity, but their cutoff values vary significantly by sex, age, and ethnicity (8). Among these and other anthropometric indicators, the waist-to-height ratio (WHtR) has emerged as the most consistent and practical alternative for assessing both central obesity and cardiometabolic risk (9) (Figure 1), and its critical boundary value of 0.5 is quite easy to communicate as “keep your waist circumference under half your height” (10). The article Research Topic gathered in this Research Topic provides a robust body of evidence supporting the usefulness of WHtR for the early identification of at-risk individuals who may benefit from preventive interventions at either the clinical or community levels.

**Figure 1:**
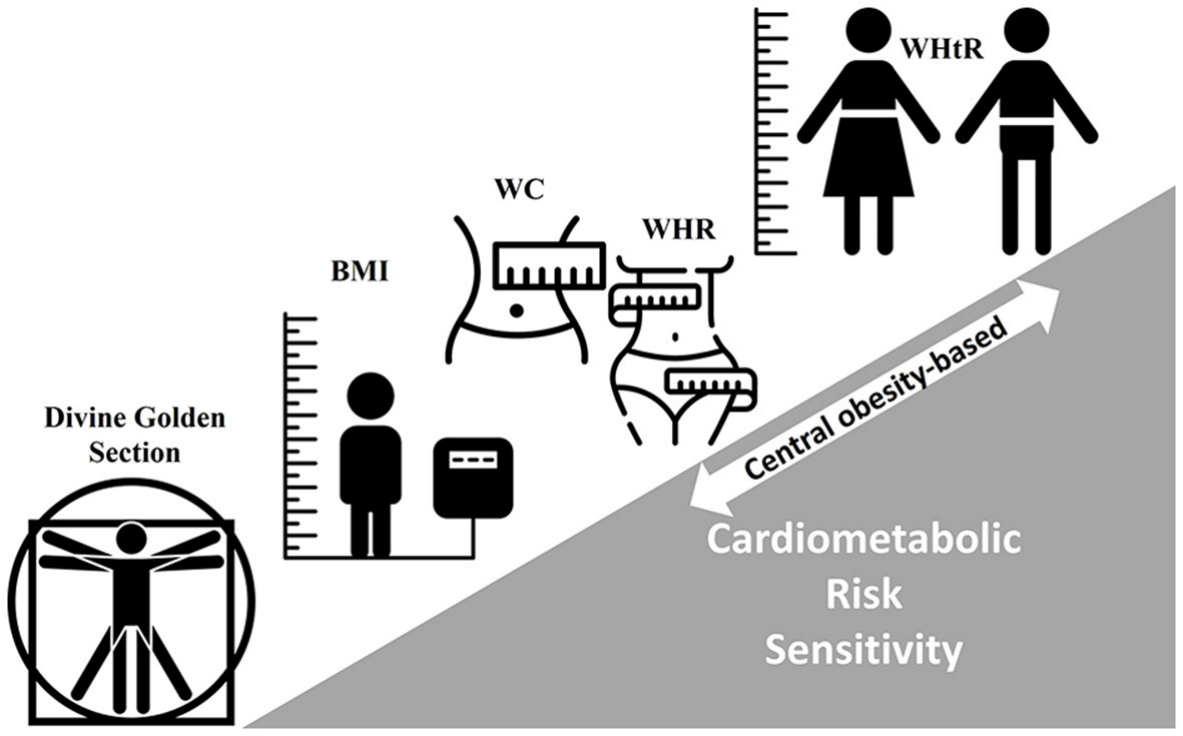
A brief history of cardiometabolic risk prediction with anthropometric indicators. Body mass index (BMI; kg.m^2^), waist circumference (WC), waist-to-hip ratio (WHR), and waist-to-height ratio (WHtR). Low-to-high cardiometabolic risk prediction sensitivity (gray scalene triangle). Source: authors.

Several studies have established WHtR as superior to BMI and WC for predicting the onset and severity of cardiometabolic conditions including hypertension, diabetes, and cardiovascular diseases in middle-aged adults (9). The population-based cross-sectional study reported by Ntimana and Choma in 791 residents (69% men, ~40 y) of a rural area of South Africa reports a crude prevalence of central obesity (WC), ~60% positively associated with gender (women > men), sociodemographic factors (unemployment, marry/widowed), and negatively with known cardiovascular risk factors (e.g., smoking and alcohol consumption). This is a peculiar finding that deserves to be explored in more depth due to its possible relationship with the hypermetabolism derived from the consumption of these substances or due to the confounding effect with the dietary intake pattern (11); whether WHtR performed better than WC in these relationships should also be studied in future investigations. Yang et al. examined the prevalence (trend overtime) of elevated WHtR and WC in adults participating in the US-NHANES (1999–2018) and its epidemiology relationship with the prevalence of various chronic non-communicable diseases. The study documented a WHtR > WC risk prediction power, a +7.9% WHtR increment in this 19 y period, and that ~25% of the studied population had a normal WC yet abnormal WHtR, facts revealing that individuals with normal WC but elevated WHtR have significantly higher odds of cardiometabolic diseases. Conversely, Fernandes et al. followed 796 non-institutionalized Brazilian seniors (94–77 y) for 9 years, documenting 25% mortality and a bi-modal (U-shape; hazard ratio = 1.5–2.0) relationship with WHtR (<0.52 or ≥0.63) and WC (<83 or ≥101 cm), the latter being particularly sensitive to CVD mortality. Since BMI, WC, and WHR correlate more closely to each other than measured total body fat, their accuracy for predicting cardiometabolic risk-related fatness in older populations is stronger than that observed in younger populations (12).

Four studies included in this Research Topic provided further evidence supporting the claim “keep your waist circumference under half your height”. Ma et al. demonstrated that a WHtR ≥ 0.52 is an early warning of health risk predictor that substitutes for age-, gender-, and race-specific WC cutoffs to diagnose metabolic syndrome [MetS; 79.4% of women (OR ≥ 3.1), 68.6% men (OR ≥ 4.82)] in 8,488 hospitalized Chinese adults (56.2% male, 56–63 y) with type II diabetes. They concluded that WHtR stands as an independent (after adjusting from metabolically relevant confounders including WC) predictor of MetS regardless of gender in T2DM patients. Guzmán-García et al. also tested the accuracy of WHtR and other anthropometric indices to detect metabolically healthy obesity (MHO, crude prevalence = 6.6–9.0%) in a convenient sample of Spanish workers (*n* = 635, 36–54 y); among the assayed anthropometric parameters, the WHtR ≤ 0.55 cutoff showed the highest discriminant capacity to detect this transient healthy condition, exhibiting a high degree of agreement (kappa = 0.81) with IDF and NCEP-ATP III criteria. Lawal et al. showed that a WHtR ≤ 0.50 has better sensitivity in predicting diabetic neuropathy (OR = 22.4) in Nigerian adults (*n* = 1,040, 58% female, 41–65) as compared to BMI and WC. Asghari et al. tracked (18.2 y) 871 Iranian adolescents (10–17 y, 49% female) through young adulthood (20–38 y) when the prevalence of high carotid intima-media thickness (cIMT) was recorded; the authors showed that WHtR outperforms BMI and WHR for predicting cIMT (particularly in pre-pubertal male people) after adjusting for adulthood anthropometric measures.

In conclusion, the studies included in this Research Topic add to the body of evidence documented in systematic reviews and meta-analyses supporting a 0.5 cutoff for primary cardiometabolic disease prevention in middle-aged individuals (9, 10, 13) while making a special scientific contribution to its usefulness for detecting subjacent conditions to established cardiometabolic disorders and for the secondary–tertiary prevention of disorders related to insulin resistance worsening. The future task is to continue documenting the validity of this (or these) cut-off point(s) in much younger populations, particularly in those of pediatric age where the presence of cardiometabolic alterations is arising early (14, 15).

## Author contributions

AW-M: Conceptualization, Investigation, Project administration, Validation, Writing—original draft, Writing—review and editing. JL: Conceptualization, Investigation, Supervision, Writing—original draft, Writing—review and editing.
